# Environmental Risk Factors for Early-Onset Alzheimer’s Dementia and Frontotemporal Dementia: A Case-Control Study in Northern Italy

**DOI:** 10.3390/ijerph17217941

**Published:** 2020-10-29

**Authors:** Giorgia Adani, Tommaso Filippini, Caterina Garuti, Marcella Malavolti, Giulia Vinceti, Giovanna Zamboni, Manuela Tondelli, Chiara Galli, Manuela Costa, Marco Vinceti, Annalisa Chiari

**Affiliations:** 1 Environmental, Genetic and Nutritional Epidemiology Research Center (CREAGEN), Department of Biomedical, Metabolic and Neural Sciences, University of Modena and Reggio Emilia, 41125 Modena, Italy; giorgia.adani@unimore.it (G.A.); tommaso.filippini@unimore.it (T.F.); caterina.garuti@gmail.com (C.G.); marcella.malavolti@unimore.it (M.M.); 2 Center for Neurosciences and Neurotechnology, Department of Biomedical, Metabolic, and Neural Sciences, University of Modena and Reggio Emilia, 41126 Modena, Italy; giulia.vinceti@unimore.it (G.V.); giovanna.zamboni@unimore.it (G.Z.); 3 Neurology Unit, Modena Policlinico-University Hospital, 41126 Modena, Italy; manuelatondelli@gmail.com (M.T.); ch.galli@ausl.mo.it (C.G.); chiari.annalisa@aou.mo.it (A.C.); 4 Nuffield Department of Clinical Neurosciences, University of Oxford, Oxford OX3 9DU, UK; 5 Primary Care Department, Modena Local Health Authority, 41124 Modena, Italy; 6 Department of Neuroscience, Psychology, Pharmacology and Child Health (NeuroFARBA), University of Florence, 50139 Florence, Italy; 7 Neurology Unit of Carpi Hospital, Modena Local Health Authority, 41012 Carpi, Italy; m.costa@ausl.mo.it; 8 Department of Epidemiology, Boston University School of Public Health, Boston, MA 02118, USA

**Keywords:** early-onset dementia, Alzheimer’s dementia, frontotemporal dementia, environmental factors, occupational factors, selenium, epidemiology, risk

## Abstract

*Background*: Early-onset dementia (EOD) is defined as dementia with symptom onset before 65 years. The role of environmental risk factors in the etiology of EOD is still undefined. We aimed at assessing the role of environmental risk factors in EOD etiology, taking into account its different clinical types. *Methods*: Using a case-control study, we recruited all EOD cases referred to Modena hospitals from 2016 to 2019, while the referent population was drawn from cases’ caregivers. We investigated residential history, occupational and environmental exposures to chemicals and lifestyle behaviors through a self-administered questionnaire. We computed the odds ratios of EOD risk (overall and restricting to the Alzheimer’s dementia (AD) or frontotemporal dementia (FTD) diagnoses) and the corresponding 95% confidence intervals using an unconditional logistic regression model. *Results*: Fifty-eight EOD patients (19 FTD and 32 AD) and 54 controls agreed to participate. Most of the investigated exposures, such as occupational exposure to aluminum, pesticides, dyes, paints or thinners, were associated with an increased odds ratio (OR) for FTD but not for AD. Long-term use of selenium-containing dietary supplements was associated with increased OR for EOD and, particularly, for FTD. For both EOD forms, smoking and playing football showed an increased odds ratio, while cycling was associated with increased risk only in FTD. Overall sports practice appeared to be a protective factor for both types. *Conclusions*: Our results suggest a role of environmental and behavioral risk factors such as some chemical exposures and professional sports in EOD etiology, in particular with reference to FTD. Overall sports practice may be associated with a reduced EOD risk.

## 1. Introduction

Early-onset dementia (EOD) is defined by the onset of dementia symptoms before the age of 65, regardless of the underlying dementia syndrome. EOD has a significant impact on patients and their families, particularly when including young children [1], as well as on patient employment and income [2]. The most frequent EOD diagnosis is Alzheimer’s dementia (EOAD) followed by frontotemporal dementia (EOFTD) or vascular dementia (VaD), based on different population studies [3,4,5].

Despite the relevance of EOD and its types, data on prevalence and incidence are scarce, and so are clues to their possible environmental and lifestyle (i.e., modifiable) risk factors [6,7]. Genetic susceptibility clearly appears to play an etiologic role for EOD, including *APP* and *PSEN1/2* gene mutations for Alzheimer’s dementia (AD) and *MAPT*, *GNR* and *C9ORF72* for frontotemporal dementia (FTD). Nevertheless, known dementia mutations may explain only a small percentage of EOD cases (<10%). Therefore, other genetic risk factors as well as environmental factors might be involved [8], and this may apply to EOD even more than late-onset dementia [9]. For this reason, with dementia, as with any other disease, the exposome model assumes an interaction between individual history of exposures and underlying genetic susceptibility of subjects, encompassing the influence of socioeconomic health determinants as well [10]. As a matter of fact, the consideration of environmental exposures as potential risk factors for dementia and other neurodegenerative diseases has considerably increased in recent years [11,12,13]. In particular, studies have focused on the role of neurotoxic agents like heavy metals and metalloids, pesticides and solvents [7,14,15,16], as well as occupational exposure [17] and air pollutants [18]. In addition, evidence of increased dementia risk associated with alcohol consumption and smoking habits has been provided, possibly through increased cardiovascular risk [6,19]. A role of traumatic brain injuries has also been suggested, although findings have been rather inconclusive [6,20]. A recent systematic review about environmental risk factors for dementia, although not specifically focused on its early-onset form, found moderate evidence of increased dementia risk associated with environmental and occupational exposure to metals and trace elements, including aluminum, lead and selenium, as well as pesticides and solvents [21]. As concerns socioeconomic components, higher educational attainment appears to reduce dementia risk [6]. Conversely, living alone seems to increase the risk compared with living with a partner [22,23].

Overall, these relations of potential etiologic relevance for dementia and, more generally, its late-onset form have not been investigated for EOD. In the present study, we aimed at investigating a broad spectrum of environmental, occupational and lifestyle factors for the two most frequent clinical EOD types, EOAD and EOFTD.

## 2. Methods

We carried out a case-control study in Modena, Northern Italy. Following approval by the Modena Ethics Committee (No. 186/2016), we recruited EOD cases referred to the Cognitive Neurology Center of the Modena Policlinico University Hospital and the Neurology Department of the Carpi Hospital from October 2016 to October 2019 [24]. Case inclusion criteria were as follows: dementia diagnosis with symptom onset before the age of 65, dementia as the principal cause of disability and residence in the province of Modena. Exclusion criteria included coexisting diagnoses of pervasive developmental disorders or major psychiatric disorders, or cognitive impairment in the context of another neurological disorder in which severe disability was caused by noncognitive symptoms (e.g., multiple sclerosis or cerebrovascular disease with severe motor disability). As a referent population, we recruited the caregivers of dementia patients irrespective of age at onset.

Each subject received a questionnaire tailored to record anamnestic and lifestyle factors potentially related to dementia onset [25,26]. The questionnaire was subdivided into sections investigating the following sets of information: (1) personal details including date and place of birth, height and weight, marital status and educational attainment; (2) clinical history such as date and place of EOD diagnosis (for cases only), history of any trauma requiring medical evaluation (with specific reference to head, trunk, arm and leg trauma) and any electric shock; (3) occupational history with a specific subsection aimed at assessing of exposure to toxic substances such as metals, pesticides and solvents, especially for occupational exposures in the agricultural sector; (4) nonoccupational activities potentially related to use of metals, pesticides and solvents or to traumatic events (i.e., sport); (5) use of dietary supplements containing selenium; smoking habits (either passive or active smoking); (6) residential history, including type of source of water supply and proximity to water bodies, industries, waste disposal sites or incinerators and overhead power lines; residential use of pesticides for plants, insects and animals/pets. The questionnaire was designed to be self-administered, allowing subjects to fill it out at home and therefore in a comfortable environment. In addition, study personnel could be contacted when the subjects had doubts about items of the questionnaire.

We used crude and adjusted multivariate unconditional logistic regression models to estimate odds ratios (ORs) and 95% confidence intervals (CIs) of EOD and its clinical types EOAD and EOFTD associated with the investigated exposures. We included sex, age (years) and educational attainment (years of education) in the multivariable model as potential confounders and effect modifiers.

## 3. Results

Out of 150 eligible subjects, 144 provided a valid address and telephone in the discharge records and could be contacted. We eventually recruited 112 participants (58 EOD patients and 54 controls), with an average response rate of 78%. The main reasons for nonparticipation were lack of time to complete the questionnaires (27/32 = 84.4%) and unwillingness to contribute to the research (5/32 = 15.6%). Clinical diagnoses of EOD patients encompassed EOAD (*N* = 32 patients, 55.2%), EOFTD spectrum (*N* = 19, 17 EOFTD and 2 progressive supranuclear palsy, 32.8%), vascular dementia (*N* = 5, 8.6%), Lewy body dementia (*N* = 1, 1.7%) and cerebral amyloid angiopathy (*N* = 1, 1.7%) (Table 1). All controls were caregivers and family members of dementia cases, including 39 (72.2%) partners, 12 (22.2%) offspring and 3 (5.5%) siblings.

The sociodemographic characteristics of study participants, including specific features for EOAD and EOFTD cases, are reported in Table 2. Among the patients, 32.8% achieved a high school level and 5.2% reached college or more. Conversely, 38.9% of the controls obtained a high school degree, while 24.4% reached college or more. Most subjects were married (82.8% and 88.9% of cases and controls, respectively), while widows/widowers were more common in cases compared to controls (10.3% vs. 1.8%). In addition, 38% of cases and 42% of controls reported never having smoked in their life.

Dementia risk according to participants’ characteristics is reported in Table 3. Women did not show higher dementia (EOD) risk overall yet exhibited a higher EOAD risk (OR 1.5, 95% CI 0.6–3.8) and lower EOFTD risk (OR 0.6, 95% CI 0.2–1.7). Conversely, age seemed not to be associated with an increased risk. Concerning educational attainment, having a high school degree or more was inversely associated with EOD risk (OR 0.4, 95% CI 0.2–0.9). Living alone (single, divorced, widowed), using only those with a stable partner as referents, was associated with increased overall EOD risk (OR 1.8, 95% CI 0.6–5.6) along with an increased EOAD risk (OR 2.4, 95% CI 0.7–8.4), whereas no change emerged for EOFTD risk (OR 0.9, 95% CI 0.2–5.5).

In Table 4, we report crude and adjusted risk estimates associated with occupational exposure to chemicals, stratified for EOAD and EOFTD clinical diagnoses. Concerning (heavy) metals, exposure to aluminum was associated with increased EOD risk, almost entirely driven by an association with EOFTD risk (OR 4.1, 95% CI 0.5–34.5). Overall pesticide occupational exposure had an OR of 2.3, with a higher risk for all three pesticide subtypes. Moreover, in this analysis, EOFTD cases almost entirely influenced the excess risk, since the OR in this group was 3.2 (95% CI 0.7–14.8) for overall pesticides (Table 4). The OR for EOD associated with overall exposure to solvents and dyes and the exposure to all subgroups was linked to increased risk, with the exception of lubricating oils. When looking at disease-specific association, exposure to these substances was linked to a much higher risk of EOFTD (OR 2.7, 95% CI 0.7–10.4). We also found a high OR in relation to the occupational use of refrigerants, antifreezes or cooling liquids. Conversely, for both EOAD and EOFTD patients, a low OR emerged for exposure to electric/electronic equipment or electromagnetic fields (Table 4). Most of the aforementioned risk estimates were statistically very unstable due to the low number of exposed subjects.

Restricting the assessment to agricultural exposure, the OR for EOD associated with pesticides further increased. A high OR for EOD was also generally found for other groups of chemicals such as fertilizers (OR 2.0, 95% CI 0.4–9.7), disinfectants (OR 2.7, 95% CI 0.2–34.8), detergents (OR 2.3, 95% CI 0.2–30.8) and oils (OR 1.7, 95% CI 0.3–8.5). All of these kinds of exposures were linked to a higher risk of EOFTD (Table 2).

As shown in Table 5, history of any trauma requiring medical evaluation before disease onset was not associated with EOD risk. Nonetheless, we found a higher value in the analysis limited to head trauma (OR 1.5, 95% CI 0.3–4.1), particularly limiting analyses to EOFTD cases (OR 3.4, 95% CI 0.8–14.6). Exposure to upper arm trauma showed an increased risk, in this case affected by EOAD cases (OR 2.2, 95% CI 0.7–6.9). Playing sports was inversely associated with disease risk (OR 0.4, 95% CI 0.2–0.9), with an equal impact for both EOFTD and EOAD patients (OR 0.3, 95% CI 0.1–1.3 and 0.4, 95% CI 0.1–1.0 respectively). This was confirmed when we limited the analysis to competitive sports only. Conversely, a higher OR emerged for two professional sports, namely football (OR 2.2, 95% CI 0.5–9.3) and cycling (OR 2.3, 95% CI 0.4–13.4). When looking at type-specific association, both sports were linked to a higher risk of EOFTD (OR 2.6, 95% CI 0.4–15.9 for football and OR 4.4, 95% CI 0.6–30.9 for cycling) compared with EOAD (OR 1.8, 95% CI 0.3–10.1 and 0.6, 95% CI 0.1–7.6 respectively). Concerning other leisure activities and habits, we found no increased risk in association with hunting, fishing and model-making. Gardening and overall pesticide use during gardening were not associated with EOD risk, while the use of fungicides during gardening was linked to a slightly increased risk, due to a markedly increased risk in EOAD patients (Table 5).

Intake of selenium-containing dietary supplements in the 20 years before recruitment yielded an OR of 2.5 (95% CI 0.9–7.0). This increased risk persisted even after excluding the use of neuroprotective supplements after disease onset (OR 1.7, 95% CI 0.6–5.0), reaching an OR of 8.7 (95 % CI 2.0–38.0) in stratified analyses for EOFTD cases. Finally, history of smoking was associated with slightly increased risk (OR 1.3, 95% CI 0.6–2.9). A similarly marginal increased risk emerged for secondhand smoke exposure (OR 1.2, 95% CI 0.5–2.9), with no difference between the two stratified groups.

ORs for residential history are reported in Table 6. Ever having lived less than 3 km from waste disposal sites was associated with an increased risk (OR 1.1, 95% CI 0.5–2.5), as was the case for having lived in the countryside or having had a farm (OR 1.8, 95% CI 0.8–4.0), along with the use of private sources (wells or fountains) of drinking water (OR 2.0, 95% CI 0.8–5.2). Finally, residential use of pesticides was associated with a generally increased risk when used for outdoor plants (OR 1.3, 95% CI 0.4–4.1), rodents and rats (OR 1.8, 95% CI 0.3–12.3) or bugs (OR 1.3, 95% CI 0.5–3.2), particularly ground bugs (OR 1.9, 95% CI 0.6–6.4) but not flying bugs (1.0, 95% CI 0.4–2.4).

## 4. Discussion

In this study, we investigated a wide spectrum of potential environmental risk factors for EOD, some of which, to the best of our knowledge, had not been previously assessed with reference to disease etiology. In our assessment, we included putative risk factors based on their neurotoxicity shown by laboratory studies, and on their association with the risk of late-onset Alzheimer’s dementia. Moreover, we selectively investigated the association of these risk factors with the two most common early-onset dementias, namely EOFTD and EOAD, hypothesizing a different etiology for these two diseases. This was a distinctive feature of our study compared with previous epidemiologic investigations.

We found evidence of a beneficial role of educational attainment for EOD prevention, consistent with what has been suggested for late-onset dementia [27]. Our results are consistent with those of a Dutch study [28], while no such association emerged in a Swedish study [29].

In our study, widow status appeared to be a risk factor for EOD. This appears to be in keeping with previous findings reporting a higher risk of developing dementia, in both early- and late-onset forms, and for subjects widowed, divorced or single [22,30], with increased likelihood for middle-aged individuals [22]. In our study, being single was less related to the development of dementia than being widowed. This could be linked to the psychological consequence of remaining alone after a partnered life, compared with never having had a stable partner. In fact, stress could impact oxidative and neurotrophic factor signaling acting on the plasticity of neural networks and favoring the mechanisms underpinning Alzheimer’s dementia [31,32].

We found an indication that history of upper arm and head trauma requiring medical evaluation may be a risk factor for EOD, albeit in different ways depending on dementia diagnosis. In particular, upper arm trauma was associated with increased EOAD risk, while head trauma was associated with EOFTD risk. This last finding is consistent with previous studies: a case-control study on 80 FTD subjects compared to 124 controls reported an OR of 3.3 (95% CI 1.3–8.1) for head trauma [33]. Moreover, a case-control study on war veterans found an association between traumatic brain injuries and dementia in FTD patients, which was stronger than in patients affected by other dementia types [34]. These results were confirmed by a recent review reporting a higher prevalence of traumatic brain injuries in FTD patients than in other dementia forms [35]. The possible underlying mechanism could depend on the induction of oxidative stress and microglial activation by trauma [36,37]. Studies on chronic traumatic encephalopathy, a progressive tauopathy occurring in sports players exposed to mild traumatic brain injuries such as concussions, for instance in American football, provide further evidence of the role of head trauma as a risk factor for dementia [38].

Concerning sports practice, we found an increased EOD risk in football (soccer) players. Such excess risk was much higher for EOFTD than for EOAD and up to twice as high in professional players compared with noncompetitive ones. Such excess risk may be due to the increased risk of head trauma characterizing that sport [39]. The increased risk linked to cycling similarly seemed to be only related to EOFTD cases, in contrast with previous studies suggesting a protective effect of cycling on global cognition [40]. Conversely, we found evidence of beneficial effects of sports in general for both EOAD and EOFTD cases. This appears to be the case particularly for swimming, even if only in association with EOAD cases. Sports practice might have a beneficial effect on late-onset dementia according to systematic reviews [41,42,43] and meta-analyses [44,45], while the association of EOD was only investigated in relation to traumatic sports [46,47]. There is some biological plausibility for a beneficial effect of sports on dementia risk, linked to positive effects on insulin and C-peptide metabolism, as recently demonstrated in subjects with a family history of diabetes [48]. Impaired glycemia and insulin resistance may predict brain amyloid deposition as well as progressive cognitive impairment in late middle-aged adults [49,50], and their reduction could therefore be beneficial against dementia etiopathogenesis.

We observed a slightly increased EOD risk associated with herbicide and fungicide use during gardening. Such excess risk was much higher for EOFTD compared with EOAD. These associations were strengthened when considering occupational exposure to chemicals including fertilizers, pesticides and other substances. Our findings are consistent with previous studies evaluating the possible association between pesticides and late-onset dementia, recently summarized in a meta-analysis which yielded a relative risk of 1.5 (95% CI 1.0–2.3) for AD at all ages [17]. Nevertheless, null results about this association in late-onset dementia have also been reported, at least for organochlorine pesticides [51].

Very few studies have assessed the role of occupational exposure to solvents and dyes, as well as refrigerants or degreasing agents, in EOD etiology [52,53,54,55]. In our study, we found an increased risk associated with occupational exposure to aluminum, although based on an extremely low number of cases and therefore statistically very imprecise. Even though there is some biological plausibility for such association [56], a study in Northern England assessing aluminum exposure through drinking water found no association with Alzheimer’s dementia, although the levels involved were much lower than potentially toxic ones [57]. Conversely, an increased risk of Alzheimer’s dementia was found to be associated with aluminum exposure both during occupational exposure and through drinking water [58]. In our study, nevertheless, the association found for overall EOD was mostly driven by excess exposure in EOFTD. This could be explained by the hypothesis of a common disease spectrum of this type of dementia with motor neuron disease, namely amyotrophic lateral sclerosis (ALS), a disease whose etiology may involve most of the risk factors we found to be associated with EOFTD [59]. In particular, some forms of FTD and ALS share the pathological substrate, the transactive DNA-binding protein 43 (TDP-43) and the *C9ORF72* gene [59,60], as well as some environmental risk factors including agricultural chemicals and professional sports involving high risk of mild traumatic brain injuries such as concussion and subconcussion [38], leading to a pro-oxidative state and consequent neuron degeneration and astrocyte dysfunction [25,61,62].

In our study, long-term use of selenium-containing dietary supplements was associated with a considerably increased risk, only partially mitigated after excluding specific “neuroprotective” supplements taken after disease onset to reduce the risk of reverse causation. If we consider only EOFTD patients, however, results were of even stronger relevance, thus reducing the risk of bias of reverse causality. While there is clear evidence that vitamin and mineral self-supplementation do not prevent cognitive decline or dementia [63,64], the possibility that some constituents of dietary supplements, namely selenium, may exert deleterious effects on cognition should also be considered, taking into account previous results in humans and the related biological plausibility [16,65].

Finally, our results seem to confirm that smoking, already found to be associated with dementia risk in some studies [66] and particularly in apolipoprotein E ε4 noncarriers [67], may be a risk factor for EOD. However, the relation between smoking and dementia has not been confirmed by some recent studies on overall dementia risk [68,69] and a meta-analysis on EOD based on three studies [70].

We acknowledge some limitations of our study. First, the number of cases and controls was limited, and the number of exposed subjects was very low for many of the environmental factors we investigated. This increased the statistical imprecision of the risk estimates and clearly hampered our ability to reliably identify “slight” causal associations. Moreover, we could not exclude the occurrence of some selection bias linked to the case-control nature of the study. This could be true to some extent for controls (caregivers), while it is less likely for cases since we tried to include all subjects referred in the study period to the two cognitive neurology clinics, the only ones providing specialized clinical care to EOD patients for the whole territory of the province. Therefore, it is unlikely that EOD patients from the underlying population of the province could not have been referred to these clinics. Since the study was based on self-completed questionnaires and long-term exposure, we also cannot entirely rule out some degree of recall bias. However, environmental risk factors for EOD are poorly recognized and even perceived not to exist in the general population, thus making the occurrence of such bias rather unlikely. Furthermore, the use of caregivers as controls could have led to some mitigation of the real association between environmental risk factors and disease risk in our study, because most of the caregivers were family members and may have shared lifestyle habits with EOD cases, thus reducing the strength of the associations we estimated. Finally, we have no availability of genetic status for the referent population, thus precluding the possibility of assessing their role in disease risk [71,72].

## 5. Conclusions

In this case-control study in a Northern Italy population, we identified positive associations between EOD and occupational exposure to aluminum, pesticides and other chemicals (dyes, paints or thinners); long-term use of selenium-containing dietary supplements; smoking; and playing football, while overall sports practice appeared to be a strong protective factor for EOD. Some of these potential etiologic factors showed different associations with disease risk when the two most common EOD forms were independently considered.

## Figures and Tables

**Table 1:** Clinical diagnosis of early-onset dementia cases.

EOD Diagnosis	*n* (%)
All EOD diagnoses	58 (100)
Alzheimer’s dementia	32 (55.2)
Frontotemporal dementia spectrum	19 (32.8)
Frontotemporal dementia	17 (29.3)
Progressive supranuclear palsy	2 (3.4)
Vascular dementia	5 (8.6)
Cerebral amyloid angiopathy	1 (1.7)
Lewy body dementia	1 (1.7)

**Table 2:** Sociodemographic characteristics of early-onset dementia cases (EOAD, early-onset Alzheimer’s dementia; EOFTD, early-onset frontotemporal dementia) and controls.

Characteristics	Cases	EOAD	EOFTD	Controls
	*n* (%)	*n* (%)	*n* (%)	*n* (%)
All subjects	58 (51.8)	32 (28.6)	19 (17.0)	54 (48.2)
Age at questionnaire filling				
Mean (SD)	65.6 (5.2)	65.8 (4.5)	65.8 (5.7)	63.8 (9.6)
<65 years	22 (37.9)	12 (37.5)	6 (31.6)	28 (51.9)
≥65 years	36 (62.1)	20 (62.5)	13 (68.4)	26 (48.2)
Age at disease onset				
Mean (SD)	59.3 (4.7)	59.7 (4.1)	59.1 (5.1)	-
Sex				
Men	25 (43.1)	11 (34.4)	11 (57.9)	23 (42.6)
Women	33 (56.9)	21 (65.6)	8 (42.1)	31 (57.4)
Educational attainment				
Primary or less	16 (27.6)	8 (25.0)	5 (26.3)	11 (20.4)
Middle school	20 (34.5)	10 (31.3)	5 (42.1)	11 (20.4)
High school	19 (32.8)	12 (37.5)	5 (26.3)	21 (38.9)
College or more	3 (5.2)	2 (6.3)	1 (5.3)	11 (20.4)
Marital status				
Married/unmarried partner	48 (82.8)	25 (78.1)	17 (89.5)	48 (88.9)
Single	1 (1.7)	1 (3.1)	-	3 (5.6)
Separated/divorced	3 (5.2)	-	2 (10.5)	2 (3.7)
Widowed	6 (10.3)	6 (18.8)	-	1 (1.8)
Smoking habits				
Ever	35 (62.5)	19 (61.3)	12 (66.7)	30 (57.7)
Never	21 (37.5)	12 (38.7)	6 (33.3)	22 (42.3)

**Table 3:** Odds ratios (OR) with 95% confidence intervals (CIs) for early-onset dementia (EOAD, early-onset Alzheimer’s dementia; EOFTD, early-onset frontotemporal dementia) related to sociodemographic characteristics.

Factors	Cases (Yes/No)	Controls (Yes/No)	OR ^1^	OR ^2^	(95% CI)
Sex (women vs. men)	33/25	31/23	0.98	0.98	(0.45–2.12)
*EOAD*	21/11	31/23	1.42	1.49	(0.59–3.81)
*FTD spectrum*	8/11	31/23	0.54	0.57	(0.19–1.70)
Age (continuous)	-	-	1.03	1.03	(0.97–1.08)
*EOAD*	-	-	1.03	1.03	(0.97–1.10)
*FTD spectrum*	-	-	1.03	1.02	(0.95–1.09)
Educational attainment					
High school or more vs. middle school or less	22/36	32/22	0.42	0.43	(0.20–0.94)
*EOAD*	14/18	32/22	0.53	0.55	(0.22–1.35)
*FTD spectrum*	6/13	21/22	0.32	0.35	(0.11–1.08)
Marital status					
Single/separated/widowed vs. married/unmarried partner	10/48	6/48	1.67	1.78	(0.56–5.59)
*EOAD*	7/25	6/48	2.24	2.43	(0.70–8.42)
*FTD spectrum*	2/17	6/48	0.94	0.91	(0.15–5.46)

^1^ Crude model; ^2^ Multivariable model adjusting for sex, age and educational attainment.

**Table 4:** Odds ratios (ORs) with 95% confidence intervals (CIs) for early-onset dementia (EOAD, early-onset Alzheimer’s dementia; EOFTD, early-onset frontotemporal dementia) related to occupational exposure to toxic agents.

Factors	Cases (Yes/No)	Controls (Yes/No)	OR ^1^	OR ^2^	(95% CI)
Occupational exposure to toxic agents	6/47	9/41	1.72	0.85	(0.22–3.29)
*EOAD*	5/26	9/41	1.14	0.60	(0.13–2.69)
*FTD spectrum*	0/19	9/41	/	/	/
Lead	5/53	6/48	0.75	0.83	(0.22–3.15)
*EOAD*	3/29	6/48	0.83	1.09	(0.23–5.18)
*EOFTD spectrum*	1/18	6/48	0.44	0.41	(0.04–4.02)
Mercury	1/57	2/52	0.46	0.31	(0.02–4.01)
*EOAD*	1/31	2/52	0.84	0.80	(0.06–10.49)
Selenium	1/57	1/53	0.93	1.56	(0.09–27.77)
*EOAD*	1/31	1/53	1.71	2.62	(0.15–46.09)
Cadmium	1/57	0/54	/	/	/
Arsenic	0/58	0/54	/	/	/
Aluminum	4/54	2/52	1.93	2.59	(0.43–15.66)
*EOAD*	1/31	2/52	0.84	1.01	(0.08–12.27)
*EOFTD spectrum*	2/17	2/52	3.06	4.11	(0.49–34.47)
Overall pesticides	9/49	5/49	1.80	2.28	(0.67–7.77)
*EOAD*	3/29	5/49	1.01	1.19	(0.25–5.58)
*EOFTD spectrum*	4/15	5/49	2.61	3.19	(0.69–14.82)
Insecticides	8/50	5/49	1.57	1.98	(0.56–6.96)
*EOAD*	2/30	5/49	0.65	0.78	(0.13–4.48)
*EOFTD spectrum*	4/15	5/49	2.61	3.19	(0.69–14.82)
Herbicides	7/51	4/50	1.72	2.36	(0.61–9.14)
*EOAD*	3/29	4/50	1.29	1.53	(0.30–7.75)
*EOFTD spectrum*	2/17	4/50	1.47	2.05	(0.31–13.62)
Fungicides	2/56	2/52	0.93	1.68	(0.21–13.31)
*EOAD*	1/31	2/52	0.84	1.44	(0.11–18.20)
*EOFTD spectrum*	0/19	2/52	/	/	/
Overall solvents and dyes	12/46	8/46	1.50	1.74	(0.61–5.02)
*EOAD*	5/27	8/46	1.06	1.40	(0.39–5.05)
*EOFTD spectrum*	6/13	8/46	2.65	2.74	(0.72–10.38)
Oil paints	5/12	3/51	2.40	2.51	(0.62–10.15)
*EOAD*	1/31	3/51	0.55	0.90	(0.08–10.04)
*EOFTD spectrum*	3/16	3/51	3.19	3.34	(0.55–20.23)
Thinner	8/50	5/49	1.57	2.07	(0.58–7.46)
*EOAD*	3/29	5/49	1.01	1.55	(0.31–7.68)
*EOFTD spectrum*	4/15	5/49	2.61	3.06	(0.63–14.82)
Paint remover	4/54	2/52	1.93	1.77	(0.30–10.56)
*EOAD*	1/31	2/52	0.84	1.02	(0.09–12.08)
*EOFTD spectrum*	2/17	2/52	3.06	2.56	(0.31–21.04)
Paints	6/52	4/50	1.44	1.34	(0.34–5.37)
*EOAD*	2/30	4/50	0.83	1.02	(0.17–6.33)
*EOFTD spectrum*	3/16	4/50	2.34	1.88	(0.35–10.10)
Adhesives	2/56	1/53	1.89	2.68	(0.22–32.63)
*EOAD*	2/30	1/53	3.53	5.24	(0.44–63.05)
Print inks and dyes	4/54	3/51	1.26	1.74	(0.35–8.72)
*EOAD*	3/29	3/51	1.76	2.39	(0.41–13.79)
*EOFTD spectrum*	1/18	3/51	0.94	1.47	(0.12–17.90)
Lubricating oils	3/55	4/50	0.68	0.80	(0.16–3.94)
*EOFTD spectrum*	3/16	4/50	2.34	2.55	(0.47–13.94)
Refrigerants, antifreezes, cooling liquids	1/57	1/53	0.93	1.73	(0.10–29.84)
*EOAD*	1/31	1/53	1.71	3.01	(0.17–54.25)
Degreasing agents	3/55	1/53	2.89	2.38	(0.23–24.40)
*EOAD*	3/29	1/53	5.48	5.52	(0.51–59.14)
Solvents (e.g., toluene, xylene)	2/56	2/52	0.93	1.04	(0.13–8.22)
*EOAD*	1/31	2/52	0.84	1.13	(0.09–13.60)
*EOFTD spectrum*	1/18	2/52	1.44	1.46	(0.11–19.43)
Dry clean products	3/55	0/54	/	/	/
Anesthetic gas	1/57	0/54	/	/	/
Electric and electronic equipment	4/54	9/45	0.37	0.54	(0.14–2.00)
*EOAD*	3/29	9/45	0.52	0.78	(0.18–3.42)
*EOFTD spectrum*	1/18	9/45	0.28	0.37	(0.04–3.53)
Electromagnetic fields	1/57	3/51	0.30	0.37	(0.04–3.81)
*EOAD*	1/31	3/51	0.55	0.71	(0.07–7.32)
Exposure during agricultural occupation					
Fertilizers	4/54	3/51	1.26	1.96	(0.39–9.74)
*EOAD*	1/31	3/51	0.55	0.70	(0.07–7.36)
*EOFTD spectrum*	2/17	3/51	2.00	3.02	(0.40–23.02)
Pesticides	7/51	3/51	2.33	3.11	(0.72–13.32)
*EOAD*	3/29	3/51	1.76	2.07	(0.38–11.41)
*EOFTD spectrum*	3/16	3/51	3.19	4.73	(0.76–29.23)
Disinfectants	2/56	1/53	1.89	2.65	(0.2–34.84)
*EOAD*	0/32	1/53	/	/	/
*EOFTD spectrum*	2/17	1/53	6.24	9.56	(0.68–135.1)
Detergents	2/56	1/53	1.89	2.26	(0.17–30.78)
*EOFTD spectrum*	2/17	1/53	6.24	8.69	(0.58–130.87)
Solvents	2/56	0/54	/	/	/
Oils	4/54	3/51	1.26	1.71	(0.34–8.48)
*EOAD*	2/30	3/51	1.13	1.27	(0.19–8.42)
*EOFTD spectrum*	2/17	3/51	2.00	3.02	(0.40–23.02)
Diesel/gasoline	4/54	4/50	0.93	1.14	(0.26–5.06)
*EOAD*	1/31	4/50	0.40	0.47	(0.05–4.58)
*EOFTD spectrum*	2/17	4/50	1.47	1.74	(0.26–11.49)
Work accident with toxicant exposure	0/58	1/53	/	/	/
Nausea/indisposition due to occupational exposure	1/57	1/53	0.93	0.98	(0.06–16.23)

^1^ Crude model; ^2^ Adjusted model for sex, age and educational attainment.

**Table 5:** Odds ratios (ORs) with 95% confidence intervals (CIs) for early-onset dementia (EOAD, early-onset Alzheimer’s dementia; EOFTD, early-onset frontotemporal dementia) according to trauma and leisure activities.

Factors	Cases (Yes/No)	Controls (Yes/No)	OR ^1^	OR ^2^	(95% CI)
Trauma that need medical evaluation	23/32	22/31	1.01	0.93	(0.42–2.07)
*EOAD*	13/18	22/31	1.02	0.99	(0.39–2.50)
*FTD spectrum*	8/9	22/31	1.25	1.17	(0.37–3.70)
Head trauma	9/46	6/47	1.53	1.54	(0.47–4.99)
*EOAD*	3/28	6/47	0.84	0.98	(0.21–4.51)
*EOFTD spectrum*	5/12	6/47	3.26	3.43	(0.80–14.60)
Trunk trauma	0/55	2/51	/	/	/
*EOAD*	0/31	2/51	/	/	/
*EOFTD spectrum*	0/17	2/51	/	/	/
Upper arm trauma	10/45	8/45	1.25	1.38	(0.47–4.03)
*EOAD*	8/23	8/45	1.96	2.16	(0.68–6.93)
*EOFTD spectrum*	2/15	8/45	0.75	0.83	(0.13–5.15)
Lower arm trauma	13/42	13/40	0.95	0.85	(0.34–2.13)
*EOAD*	9/22	13/40	1.26	1.13	(0.40–3.19)
*EOFTD spectrum*	3/14	13/40	0.66	0.51	(0.12–2.20)
Electric shock/trauma	1/57	2/52	0.46	0.18	(0.01–2.22)
*EOAD*	0/32	2/52	/	/	/
*EOFTD spectrum*	0/19	2/52	/	/	/
Leisure activities					
Hunting	2/56	2/52	0.93	1.03	(0.13–8.25)
*EOFTD spectrum*	2/17	2/52	3.06	2.92	(0.33–25.90)
Fishing	6/52	7/47	0.77	0.65	(0.18–2.35)
*EOAD*	1/31	7/47	0.22	0.19	(0.02–1.82)
*EOFTD spectrum*	4/15	7/47	1.79	1.20	(0.25–5.65)
Painting	7/47	6/46	1.14	1.08	(0.32–3.66)
*EOAD*	4/26	6/46	1.18	1.28	(0.30–5.49)
*EOFTD spectrum*	3/14	6/46	1.64	1.60	(0.31–8.15)
Model-making	3/55	3/51	0.93	1.11	(0.19–6.42)
*EOAD*	1/31	3/51	0.55	0.71	(0.06–8.02)
*EOFTD spectrum*	2/17	3/51	2.00	2.21	(0.28–17.20)
Gardening	22/36	26/28	0.66	0.71	(0.32–1.57)
*EOAD*	13/19	26/28	0.74	0.87	(0.34–2.24)
*EOFTD spectrum*	6/13	26/28	0.50	0.48	(0.15–1.55)
Using pesticides during gardening	7/51	8/46	0.79	0.84	(0.27–2.55)
*EOAD*	4/28	8/46	0.82	0.84	(0.23–3.11)
*EOFTD spectrum*	2/17	8/46	0.68	0.63	(0.11–3.49)
Using herbicides during gardening	4/54	4/50	0.93	1.31	(0.28–6.08)
*EOAD*	2/30	4/50	0.83	1.10	(0.17–7.07)
*EOFTD spectrum*	1/18	4/50	0.69	1.13	(0.10–13.45)
Using fungicides during gardening	6/52	4/50	1.44	1.56	(0.40–6.10)
*EOAD*	5/27	4/50	2.31	2.95	(0.67–13.0)
*EOFTD spectrum*	1/18	4/50	0.69	0.58	(0.06–6.12)
Developing pictures in darkroom	1/57	1/53	0.93	1.00	(0.06–17.79)
*EOAD*	1/31	1/53	1.71	2.21	(0.12–14.15)
Playing sport	15/43	28/26	0.32	0.36	(0.15–0.89)
*EOAD*	8/24	28/26	0.31	0.37	(0.13–1.04)
*EOFTD spectrum*	5/14	28/26	0.33	0.34	(0.09–1.26)
Playing competitive sport	4/54	8/46	0.43	0.45	(0.11–1.74)
*EOAD*	1/31	8/46	0.19	0.19	(0.02–1.75)
*EOFTD spectrum*	3/16	8/46	1.08	1.05	(0.21–5.15)
Football	7/51	4/50	1.72	2.23	(0.54–9.26)
*EOAD*	3/29	4/50	1.29	1.78	(0.32–10.06)
*EOFTD spectrum*	3/16	4/50	2.34	2.62	(0.43–15.90)
Volleyball	2/56	6/48	0.29	0.36	(0.07–1.92)
*EOAD*	1/31	6/48	0.26	0.31	(0.03–2.80)
*EOFTD spectrum*	1/18	6/48	0.44	0.66	(0.07–6.20)
Cycling	5/53	2/52	2.45	2.28	(0.39–13.43)
*EOAD*	1/31	2/52	0.84	0.60	(0.05–7.56)
*EOFTD spectrum*	3/16	2/52	4.87	4.37	(0.62–30.93)
Swimming	2/56	12/42	0.13	0.17	(0.03–0.84)
*EOAD*	2/30	12/42	0.23	0.28	(0.06–1.45)
Athletics	2/56	6/48	0.29	0.29	(0.05–1.56)
*EOAD*	1/31	6/48	0.26	0.24	(0.03–2.13)
*EOFTD spectrum*	1/18	6/48	0.44	0.49	(0.05–4.74)
Running/trekking	1/57	4/50	0.22	0.34	(0.03–3.25)
*EOFTD spectrum*	1/18	4/50	0.69	1.13	(0.11–12.09)
Use of dietary supplements containing selenium in the past 20 years	14/44	8/46	1.83	2.50	(0.89–7.02)
*EOAD*	6/26	8/46	1.33	2.01	(0.57–7.10)
*EOFTD spectrum*	7/12	8/46	3.35	7.40	(1.65–33.19)
Excluding specific neuroprotective supplements after onset of disease	11/47	8/46	1.35	1.73	(0.60–4.99)
*EOAD*	3/29	8/46	0.59	0.78	(0.18–3.41)
*EOFTD spectrum*	8/11	8/46	4.18	8.73	(2.01–38.00)
Use of selenized potatoes	7/51	11/43	0.54	0.55	(0.19–1.62)
*EOAD*	3/29	11/43	0.40	0.40	(0.10–1.63)
*EOFTD spectrum*	3/16	11/43	0.73	0.70	(0.16–3.05)
Ever smoking	35/23	30/24	1.22	1.28	(0.58–2.86)
*EOAD*	19/13	30/24	1.17	1.35	(0.51–3.55)
*EOFTD spectrum*	12/7	30/24	1.37	1.31	(0.42–4.09)
Current smoking	10/48	10/44	0.92	1.01	(0.37–2.79)
*EOAD*	5/27	10/44	0.81	0.87	(0.25–2.99)
*EOFTD spectrum*	3/16	10/44	0.83	1.09	(0.24–4.89)
Passive smoking exposure	15/43	12/42	1.22	1.17	(0.47–2.88)
*EOAD*	9/23	12/42	1.37	1.24	(0.44–3.46)
*EOFTD spectrum*	5/14	12/42	1.25	1.22	(0.33–4.50)

^1^ Crude model; ^2^ Adjusted model for sex, age and educational attainment.

**Table 6:** Odds ratios (ORs) with 95% confidence intervals (CIs) for early-onset dementia (EOAD, early-onset Alzheimer’s dementia; EOFTD, early-onset frontotemporal dementia) related to residential history.

Factors	Cases (Yes/No)	Controls (Yes/No)	OR ^a^	OR ^b^	(95% CI)
Residential history					
Ever lived in the countryside or had a farm	27/31	21/33	1.37	1.79	(0.80–4.03)
*EOAD*	15/17	21/33	1.39	1.54	(0.61–3.90)
*FTD spectrum*	8/11	21/33	1.14	1.68	(0.52–5.41)
Ever used private well or a fountain for drinking water	16/42	11/43	1.49	2.04	(0.80–5.21)
*EOAD*	9/23	11/43	1.53	2.14	(0.71–6.47)
*EOFTD spectrum*	5/14	11/43	1.40	1.86	(0.50–6.93)
Ever used private well or fountain for irrigation	5/53	10/44	0.42	0.46	(0.14–1.48)
*EOAD*	1/31	10/44	0.14	0.17	(0.02–1.41)
*EOFTD spectrum*	2/17	10/44	0.52	0.54	(0.10–2.89)
Ever lived less than 3 km from water bodies	25/33	24/30	0.95	1.14	(0.52–2.52)
*EOAD*	14/18	24/30	0.97	1.11	(0.44–2.81)
*EOFTD spectrum*	6/13	24/30	0.58	0.69	(0.22–2.17)
Having lived near:					
Waste incinerator	1/57	3/51	0.30	0.28	(0.03–2.99)
*EOAD*	1/31	3/51	0.55	0.49	(0.05–5.13)
Waste disposal site	4/54	3/51	1.26	1.88	(0.38–9.40)
*EOAD*	3/29	3/51	1.76	2.54	(0.45–14.52)
*EOFTD spectrum*	1/18	3/51	0.94	1.46	(0.12–17.19)
Toxic plant/industry	11/47	14/40	0.67	0.63	(0.25–1.61)
*EOAD*	5/27	14/40	0.53	0.51	(0.16–1.62)
*EOFTD spectrum*	4/15	14/40	0.76	0.68	(0.18–2.55)
Overhead power lines	7/51	11/43	0.54	0.67	(0.23–1.99)
*EOAD*	2/30	11/43	0.26	0.28	(0.06–1.39)
*EOFTD spectrum*	3/16	11/43	0.73	1.04	(0.23–4.58)
Residential use of pesticides	16/42	18/36	0.76	1.00	(0.42–2.35)
*EOAD*	7/25	18/36	0.56	0.68	(0.24–1.98)
*EOFTD spectrum*	5/14	18/36	0.71	0.87	(0.26–2.94)
By substrate:					
Plants	7/51	8/46	0.79	0.92	(0.30–2.84)
*EOAD*	2/30	8/46	0.38	0.40	(0.08–2.06)
*EOFTD spectrum*	3/16	8/46	1.08	1.50	(0.33–6.90)
Outdoor plants	7/51	6/48	1.10	1.25	(0.38–4.10)
*EOAD*	2/30	6/48	0.53	0.56	(0.10–3.02)
*EOFTD spectrum*	3/16	4/48	1.50	1.97	(0.41–9.45)
Indoor plants	2/56	4/50	0.45	0.58	(0.10–3.56)
*EOAD*	1/31	4/50	0.40	0.45	(0.04–4.53)
*EOFTD spectrum*	1/18	4/50	0.69	1.08	(0.10–11.62)
Bugs	15/43	14/40	1.00	1.29	(0.53–3.16)
*EOAD*	8/24	14/40	0.95	1.26	(0.43–3.70)
*EOFTD spectrum*	4/15	14/40	0.76	0.86	(0.23–3.25)
Flying bugs	12/46	14/40	0.75	0.97	(0.38–2.44)
*EOAD*	6/26	14/40	0.66	0.85	(0.27–2.64)
*EOFTD spectrum*	3/16	14/40	0.54	0.66	(0.16–2.76)
Ground bugs	8/50	6/48	1.28	1.89	(0.56–6.40)
*EOAD*	5/27	6/48	1.48	2.13	(0.54–8.47)
*EOFTD spectrum*	2/17	6/48	0.94	1.24	(0.20–7.67)
Rodents and rats	3/55	2/52	1.42	1.83	(0.27–12.26)
*EOAD*	2/30	2/52	1,73	1.99	(0.24–16.28)
*EOFTD spectrum*	1/18	2/52	1.44	2.24	(0.16–31.13)
Pets	8/50	11/43	0.63	0.77	(0.27–2.17)
*EOAD*	3/29	11/43	0.40	0.44	(0.11–1.81)
*EOFTD spectrum*	4/15	11/43	1.04	1.23	(0.32–4.76)
Municipal pesticides use	22/36	21/33	0.96	1.05	(0.47–2.33)
*EOAD*	13/19	21/33	1.08	1.22	(0.48–3.09)
*EOFTD spectrum*	7/12	21/33	0.92	0.91	(0.30–2.80)

^a^ Crude model; ^b^ Adjusted model for sex, age and educational attainment.
